# First report of myiasis caused by *Cochliomyia hominivorax* in free-ranging giant otter (*Pteronura brasiliensis*)

**DOI:** 10.1590/S1984-29612022058

**Published:** 2022-11-21

**Authors:** Nathalie Foerster, Grazielle Soresini, Fernando Paiva, Fabiano Aguiar da Silva, Caroline Leuchtenberger, Guilherme Mourão

**Affiliations:** 1 Programa de Pós-Graduação Em Ecologia e Conservação, Universidade Federal de Mato Grosso do Sul, Campo Grande, MS, Brasil; 2 Giant Otter Conservation Fund, Arroio do Meio, RS, Brasil; 3 Instituto de Biociências, Universidade Federal de Mato Grosso do Sul, Campo Grande, MS, Brasil; 4 Instituto Federal Farroupilha, Santa Maria, RS, Brasil; 5 Laboratório de Vida Selvagem, Embrapa Pantanal, Corumbá, MS, Brasil

**Keywords:** Mustelid, ectoparasite, Lutrinae, Pantanal, neotropical wetland, Mustelídeo, ectoparasito, Lutríneos, Pantanal, áreas úmidas neotropicais

## Abstract

Giant otters are territorial semi-aquatic mammals. It is common to find several individuals exhibiting wounds and scars due to intraspecific conflicts. Myiasis is a parasitic infestation on living tissues of vertebrates caused by dipterous larvae, that usually develops in freshly open wounds and can seriously threaten the host’s health. Ectoparasites seem to be rare among giant otters and myiasis had not been recorded in this species until now. Here, is presented one record of myiasis in a free-ranging giant otter found dead in the Pantanal, Brazil. An ulcerative lesion was found in the frontoparietal region, from which 22 larvae were recovered and identified as *Cochliomyia hominivorax.* The low occurrence of ectoparasites in giant otters might reflect their semi-aquatic habits and their grooming behavior, which makes it difficult for parasites to remain on the skin. The injured otter probably got the larvae after an intraspecific fight. Agonistic encounters between groups of giant otters have been reported before and these fights can result in serious wounds or even death. It was hypothesized that the myiasis caused by *C. hominivorax* deteriorated the health of the infested giant otter, which prevented recovery and accelerated its death.

## Resumo

As ariranhas são mamíferos semiaquáticos habitantes do território brasileiro. É comum encontrar vários indivíduos apresentando feridas e cicatrizes devido a conflitos intraespecíficos. A miíase é uma infestação parasitária em tecidos vivos de vertebrados, causada por larvas de dípteros que, geralmente, se desenvolvem em feridas recém-abertas, podendo ameaçar seriamente a saúde do hospedeiro. Ectoparasitos parecem ser raros em ariranhas e, até o presente, não há relatos de miíase nesta espécie. Aqui, é apresentado um registro de miíase em uma ariranha de vida livre, encontrada morta no Pantanal, Brasil. Foi encontrada uma lesão ulcerativa na região frontoparietal, na qual foram recuperadas 22 larvas identificadas como *Cochliomyia hominivorax*. A baixa ocorrência de ectoparasitos em ariranhas pode refletir seus hábitos semiaquáticos e seu comportamento de limpeza, o que dificulta a permanência dos parasitos na pele. A ariranha ferida, provavelmente, se infestou com as larvas após uma briga intraespecífica. Encontros agonísticos entre grupos de ariranhas já foram relatados antes, e essas lutas podem resultar em ferimentos graves ou até mesmo em morte. Foi hipotetizado que a miíase causada por *C. hominivorax* deteriorou a saúde da ariranha infestada, o que impediu a recuperação e acelerou sua morte.

## Introduction

Giant otters (*Pteronura brasiliensis*) are semi-aquatic mammals and the largest members of the family Mustelidae. They are social and territorial. Giant otters scent-mark and vocalize a wide repertoire to mark their territories and to avoid agonistic encounters with other groups (Leuchtenberger & Mourão, 2009). However, during the dry season their territories shrink, and the conflicts tend to increase (Ribas & Mourão, 2004; Leuchtenberger & Mourão, 2009), and it is common to find several individuals exhibiting wounds and scars (Rosas & Mattos, 2003).

Myiasis is a parasitic infestation on living or necrotic tissues of vertebrates caused by dipterous larvae (Hope, 1837; Zumpt, 1965). Myiasis can involve heavy infestation of freshly open wounds causing swelling, inflammation, pain, and thus seriously threat the host’s health (Hall, 1997; Yan et al., 2019). The North American river otter (*Lontra canadensis*) is susceptible to myiasis (Kimber & Kollias, 2000). However, it was unknown if the giant otter was susceptible to myiasis.

There are a few records of myiasis in free-ranging mammals in Brazil, including in maned wolf (*Chrysocyon brachyurus*) (Cansi et al., 2011), porcupine (*Coendou prehensilis*) (Lacey & George, 1981), opossum (*Didelphis marsupialis*) (Reis et al., 2008) and gracile mouse opossum (*Gracilinanus* sp.) (Reis et al., 2008 ). Recently, May-Júnior et al. (2021) captured 13 jaguars in the Pantanal presenting subcutaneous nodules due to parasitism by *Dermatobia hominis* larvae. In some of these jaguars, myiasis caused by *Cochliomyia hominivorax* was also found. In mustelids, only two records of myiasis infestation have been reported in Brazil. One occurred in a captive lesser grison (*Galictis cuja*) (Figueiredo et al., 2010), and the other in a neotropical otter (*Lontra longicaudis*) that was rescued exhibiting health problems on the banks of a lake in southern Brazil (Michelazzo et al., 2022). A report of myiasis in the North American river otter resulted in death three days after it was captured due to extensive damage caused by the dipteran larvae, which were not specifically identified (Serfass et al., 1993).

Here, is presented one record of myiasis caused by the larval stage of *Cochliomyia hominivorax*, in a free-ranging giant otter in the Pantanal, a large wetland located near the center of South America. To the author’s knowledge, this is the first report of myiasis in giant otters.

## Methods

On the morning of September 4, 2021, a dead giant otter was found floating at the side of the Miranda River (19° 31' 13.95” S 57° 7' 12.96” W), in the state of Mato Grosso do Sul, Brazil. The animal was near the entrance of a former den. The carcass was collected and taken straight to a field lab (license SISBIO/ICMBio 79173-1). Judging from the fresh condition of the carcass, the time of death was estimated as only a few hours before the animal was found.

**Figure 1:**
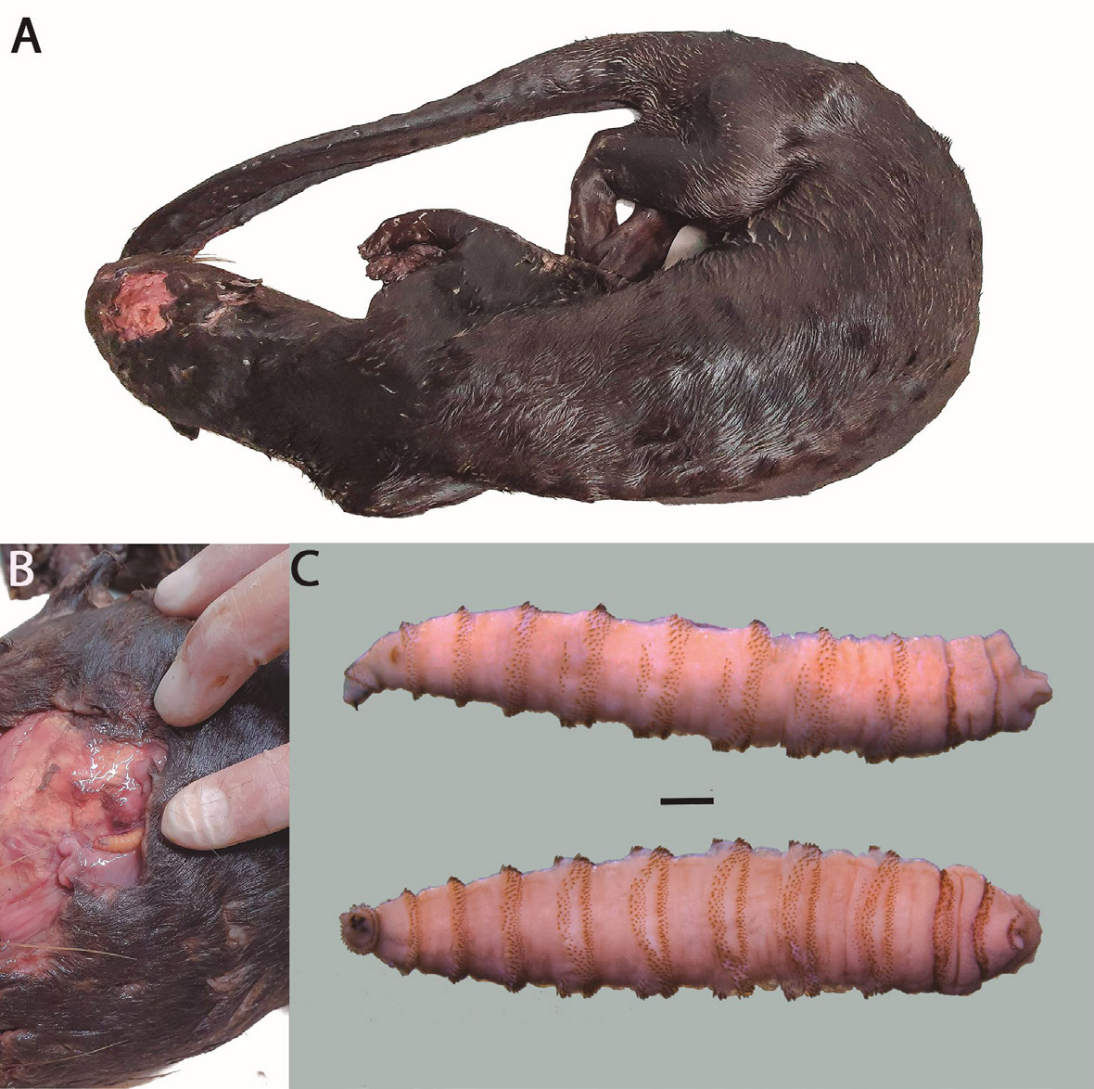
Carcass of a giant otter (*Pteronura brasiliensis*) found in the wild with myiasis lesion. A - General view of the animal; B - Details of the lesion, with third-instar larva; C - Third-instar larva of *Cochliomyia hominivorax* (Coquerel, 1858) recovered from skin; lateral view and ventral view (bar = 1 mm).

It was a young male, in poor body condition, measuring 116 cm in total length and weighing 18.77 kg. This individual had several injuries along its body, most of them probably due to bites and other wounds from a possible fight (Figure 1A and 1B). At the necropsy, it was observed that the animal no longer had any fat tissue remaining and the internal organs had normal macroscopic appearance. In a skin lesion measuring 8.5 x 6 cm, in the frontoparietal region with many cavitations on the edges, dipteran larvae were found. All the larvae were collected and stored in 70° GL ethyl alcohol for subsequent identification.

The larvae were cleaned with the aid of a brush and then were examined for their taxonomic characteristics by means of light microscopy, under a Leica M205 C™ stereomicroscope or a Leica DM5500 B™ microscope, both equipped with Leica cameras, models DFC 420 and 490, respectively (Leica Microsystems™, Wetzlar and Mannheim, Germany). Images were registered in the Leica Application Suite image analysis system (LAS™ 3.8; Leica Microsystems™, Wetzlar and Mannheim, Germany). Two specimens were randomly selected and passed through a clarification process, using a solution of potassium hydroxide (KOH) (10% w/v) (Zumpt, 1965), and were placed in an oven at 46°C until translucent (approximately five hours). The specimens were dehydrated in a progressive ethyl alcohol series, from 70 to 99° GL, at one-hour intervals between each dilution (70, 80, 90 and 99° GL). Then they were immersed in hexamethyldisilazane (cat. number 440191; Sigma-Aldrich™) for 10 minutes, followed by deposition onto carbon conductive tabs (12 mm OD, Pelco Tabs™; Ted Pella®, Inc., USA) attached to Pelco® Q pin stubs of dimensions 12.7 × 12.7 mm (Ted Pella®, Inc., USA). The images were documented using a Hitachi® model TM3000™ scanning electron microscope (Hitachi, Tokyo, Japan) in the analysis mode. The specimens were deposited in the Zoological Reference Collection of the Federal University of Mato Grosso do Sul (ZUFMS-DIP01276).

The taxonomic characteristics considered were those previously described by Laake et al. (1936), Knipling (1939), James (1947), Zumpt (1965) and Shewell (1981).

## Results and Discussion

During the necropsy examination of the giant otter 22 live larvae were recovered. Macroscopically, the larvae were whitish, cylindrical, and tapered anteriorly, with 12 visible segments surrounded by band spines (Figure 1C). The lesion was characterized as typical primary ulcerative myiasis (Laake et al., 1936; Knipling, 1939; James, 1947).

**Figure 2:**
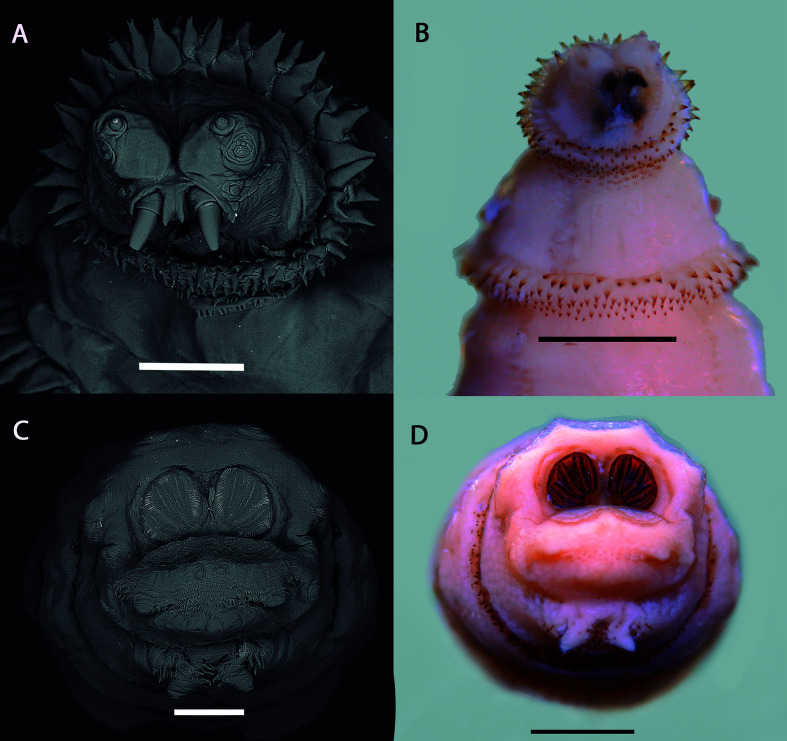
Third-instar larva of *Cochliomyia hominivorax* (Coquerel, 1858) recovered from skin lesion in giant otter (*Pteronura brasiliensis*) in the Pantanal, Brazil. A and B - Details of the anterior portion; C and D - View of the posterior portion. Scale bars: A and C = 250 µm; B and D = 1 mm.

**Figure 3:**
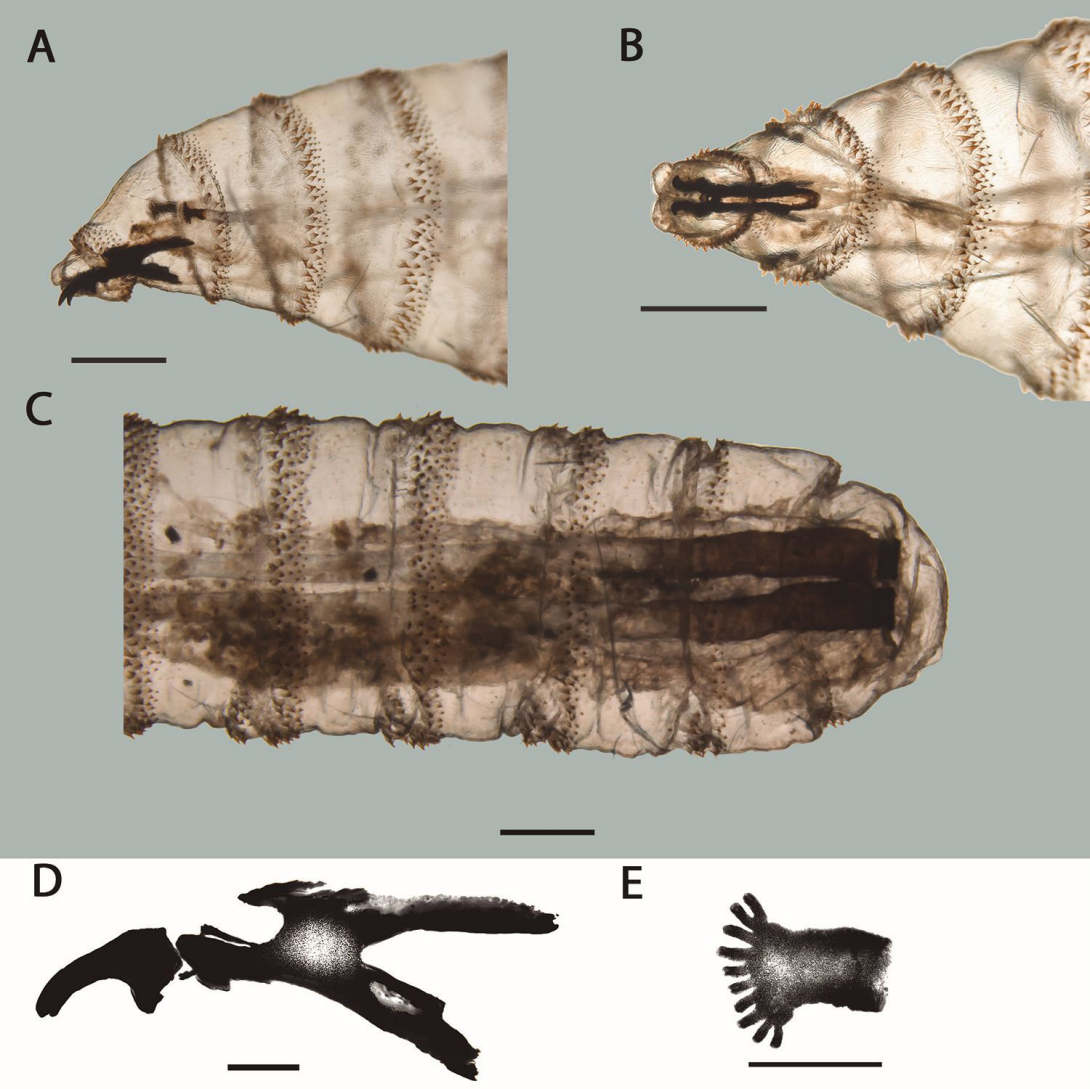
Third-instar larva of *Cochliomyia hominivorax* (Coquerel, 1858) recovered from skin lesion in giant otter (*Pteronura brasiliensis*) in the Pantanal, Brazil. A, B and C - specimen cleared with 10% solution of potassium hydroxide (KOH) (bar = 1 mm); A - Anterior portion in lateral view; B - Anterior portion in ventral view; C - Posterior portion in ventral view, highlighting the pigmentation of the tracheal trunks. D - Diagrammatic drawing of cephalopharyngeal apparatus in the lateral view (bar = 200 µm); and E - Diagrammatic drawing of anterior spiracle (bar = 250 µm).

All the larvae were in the third stage and were identified as *Cochliomyia hominivorax* (Coquerel, 1858). They showed the following characteristics: anterior portion armed with a pair of strong mouth hooks (Figure 2A and 2B); peritremes of posterior spiracle incomplete and not defined enclosing the button poorly; encircling three straight subparallel opening lined up diagonally (Figure 2C and 2D); cephalopharyngeal apparatus well developed and highly sclerotized, with conspicuous paired of mandibular hooks (Figure 3A and 3B); tracheal trunks lightly blackened, pigmented from the posterior spiracle (Figure 3C); dorsal region of the cornua not incised (Figure 3D); and anterior spiracle with short flattened stalk, with 10 nodular branches arranged fanwise (Figure 3E) (Knipling, 1939; James, 1947; Shewell, 1981). The length of the larvae ranged from 10 to 15 mm.

*Cochliomyia hominivorax*, commonly known as the New World screwworm (NWS), is a dipteran species of the family Calliphoridae. The NWS is one of the main causes of myiasis in livestock, wildlife, and humans in tropical and subtropical parts of the Americas where it has not been eradicated, including Brazil (Wyss, 2000; Zumpt, 1965). Gravid adult female *C. hominivorax* lay their eggs in open wounds on the host. Upon hatching, the fly larvae, or maggots, also known as screwworms, feed on living dermal or subdermal tissues of the parasitized host (Knipling, 1939).

Myiasis is very rare in aquatic vertebrates, with only a few records in fish (Bristow et al., 1990; Öktener & Alas, 2009; Zumpt, 1965). The low occurrence of ectoparasites in giant otters, especially dipterans, might reflect their semi-aquatic habits, but also their grooming and allogrooming behavior, which makes it difficult for parasites to remain on the skin.

The NSW cannot develop in carrion; it feeds only on living tissues. The time taken for the larvae to reach the third stage is 5 to 7 days (Hall, 1997). Thus, the otter probably acquired the NSW after being injured either by an accident or even intraspecific fights. Agonistic encounters between groups of giant otters have been reported before (Ribas & Mourão, 2004). These fights can result in serious wounds or even death (Rosas & Mattos, 2003; Leuchtenberger et al., 2015), even though many individuals can show rapid recovery from injuries in the wild (Foerster, person. obs.). It was hypothesized that the myiasis caused by *C. hominivorax* deteriorated the health of the infested giant otter, which prevented recovery and accelerated its death.

The NSW is very important in economic terms where endemic, since it infests cattle and other livestock species (Vargans-Terán, 2020). In Brazil, the economic loss caused by NWS infestation on the livestock industry was estimated to be USD 380 million annually (Grisi et al., 2014). Although information regarding its impact on wildlife populations is scant, Vargas-Terán (1991) reported an 80% loss among fawns of the white-tailed deer (*Odocoileus virginianus*) in Texas, in the United States, duet to NSW infestation.

The records of myiasis in endangered species such as jaguars (May-Junior, 2021), giant otters reported herein, and invasive vertebrate species like feral hogs (Altuna et al., 2021), demonstrate the importance of studies on the impact of these parasites on wildlife.
